# Effects of MICU1-Mediated Mitochondrial Calcium Uptake on Energy Metabolism and Quality of Vitrified-Thawed Mouse Metaphase II Oocytes

**DOI:** 10.3390/ijms23158629

**Published:** 2022-08-03

**Authors:** Tianyang Lan, Kang Zhang, Feifei Lin, Qifu He, Shenghui Wu, Zhiming Xu, Yong Zhang, Fusheng Quan

**Affiliations:** Key Laboratory of Animal Biotechnology of the Ministry of Agriculture, Northwest A&F University, Yangling 712100, China; lty1357121@163.com (T.L.); zhangk3581@163.com (K.Z.); linfeifei0126@126.com (F.L.); hqf1024456192@163.com (Q.H.); wushenghui@nwafu.edu.cn (S.W.); 13592572562@nwafu.edu.cn (Z.X.)

**Keywords:** MICU1, mitochondria, oocyte vitrification, pyruvate dehydrogenase

## Abstract

Background: Oocyte vitrification has been widely used in the treatment of infertility and fertility preservation. However, vitrification-induced mitochondrial damage adversely affects oocyte development. Several studies have reported that mitochondrial calcium uptake protein 1 (MICU1) regulates the uptake of mitochondrial calcium by the mitochondrial calcium uniporter (MCU) and subsequently controls aerobic metabolism and oxidative stress in mitochondria, but research considering oocytes remains unreported. We evaluated whether the addition of MICU1 modulators enhances mitochondrial activity, pyruvate metabolism, and developmental competence after warming of MII oocytes. Methods: Retrieved MII oocytes of mice were classified as vitrified or control groups. After thawing, oocytes of vitrified group were cultured with or without DS16570511 (MICU1 inhibitor) and MCU-i4 (MICU1 activator) for 2 h. Results: Mitochondrial Ca^2+^ concentration, pyruvate dephosphorylation level, and MICU1 expression of MII oocytes were significantly increased after vitrification. These phenomena were further exacerbated by the addition of MCU-i4 and reversed by the addition of DS16570511 after warming. However, the mitochondrial membrane potential (MMP) and adenosine triphosphate (ATP) in vitrified-warmed MII oocytes drop significantly after vitrification, which was improved after MCU-i4 treatment and decreased significantly after DS16570511 treatment. The vitrification process was able to elicit a development competence reduction. After parthenogenetic activation, incubation of the thawed oocytes with MCU-i4 did not alter the cleavage and blastocyst rates. Moreover, incubation of the thawed oocytes with DS16570511 reduced the cleavage and blastocyst rates. Conclusions: MICU1-mediated increasing mitochondrial calcium uptake after vitrification of the MII oocytes promoted the pyruvate oxidation, and this process may maintain oocyte development competence by compensating for the consumption of ATP under stress state.

## 1. Introduction

Vitrified-thawed oocytes provide abundant experimental materials for embryo biotechnology, such as artificial fertilization and somatic cell clones, thus contributing significantly to assisted reproduction and genetic resource conservation [1]. Compared with programmed cryopreservation, vitrification demonstrates the advantages of reduced ice crystal formation, minimal cryoinjury, and a relatively easy to perform with a wide range of applications [2]. However, vitrification can seriously damage the mitochondrial function of oocytes and adversely affect their developmental potential after thawing. This may occur through decreased membrane potential, and lower ATP content [3,4].

Mitochondrial Ca^2+^ regulation is crucial for mitochondrial function [5,6]. Mitochondrial calcium uptake protein 1 (MICU1) is an important regulatory subunit of the mitochondrial calcium uniporter (MCU) located in the inner membrane of mitochondria [7]. It senses and bidirectionally regulates cytoplasmic Ca^2+^ concentration [8,9]. Specifically, MICU1 can act as a gatekeeper for MCU-mediated Ca^2+^ uptake by setting a threshold to prevent Ca^2+^ uptake when cytoplasmic calcium levels are low and the threshold is not reached [10]. Next, it may function as an activator to coordinate with MCU to promote Ca^2+^ uptake when intracytoplasmic Ca^2+^ levels rise to a threshold without affecting the dynamic properties of MCU-mediated Ca^2+^ uptake [11]. Therefore, MICU1 inhibits excessive accumulation of intracellular Ca^2+^ and the production of reactive oxygen species (ROS), and it protects cells from calcium overload and subsequent cell damage [10]. In addition, MICU1 demonstrates a unique role in regulating glucose metabolism in ovarian cancer cells by shifting the mitochondrial glucose metabolism (tricarboxylic acid cycle) pathway to aerobic glycolysis for tumor growth and drug resistance [12]. However, the role of MICU1 in the vitrification of oocytes has not been elucidated. In this study, we determined the effect of MICU1 on mitochondrial Ca^2+^ concentration, to determine whether MICU1 is involved in the recovery of mitochondrial metabolism after oocyte thawing by affecting pyruvate dehydrogenase (PDH).

## 2. Results

### 2.1. Effects of Oocyte Vitrification on Mitochondrial Ca^2+^ Uptake

**Figure 1:**
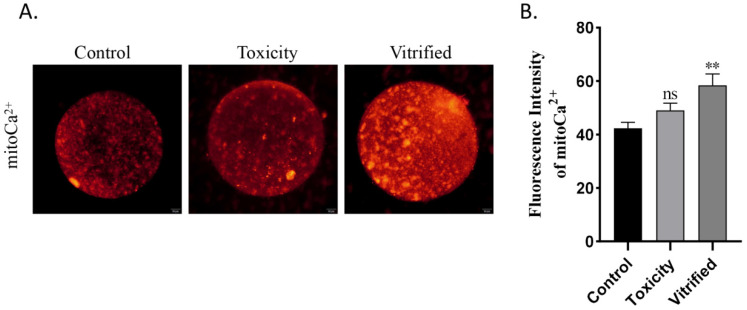
Oocyte vitrification increases mitochondrial Ca^2+^ uptake. (**A**) Fluorescence images showing the mitochondrial Ca^2+^ levels of oocytes in each group. Scale bar = 20 μm. (**B**) Data of mitochondrial Ca^2+^ fluorescence intensity as presented as the mean ± SEM. of 3 to 5 independent experiments per group. ns, no significant, ** *p* < 0.01.

To determine whether vitrification affects mitochondrial Ca^2+^ uptake, we used Rhod-2 AM, a Ca^2+^ fluorescence probe that specifically aggregates in mitochondria because of its positive charge, to track mitochondrial Ca^2+^ uptake in oocytes. The fluorescence intensity of Rhod-2 AM in the vitrified group was significantly increased compared with that in the control groups (*p* < 0.01; Figure 1). This indicates that mitochondrial Ca^2+^ concentration was significantly increased in vitrified oocytes. In the toxicity groups, the fluorescence intensity of Rhod-2 was unaffected compared with the control groups (*p* > 0.05; Figure 1). These results suggest that oocyte vitrification accelerated the uptake of Ca^2+^ by the mitochondria (Figure 1).

### 2.2. MICU1 Is Upregulated in Vitrified Oocytes

**Figure 2:**
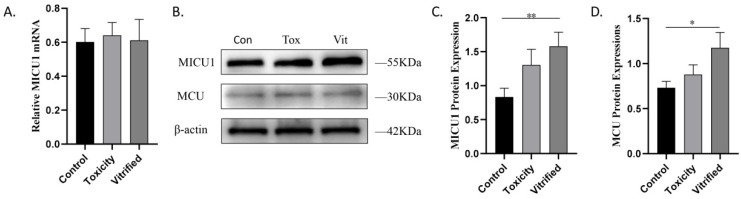
Effect of vitrification on the mRNA of MICU1 (**A**), the protein of MICU1 (**B**,**C**) and MCU (**B**,**D**) expressions in oocytes. Data are presented as the mean ± SEM. of 3 to 5 samples per group. * *p* < 0.05, ** *p* < 0.01. Con, control; Tox, toxicity; Vit, vitrified.

MICU1 is located in the inner mitochondrial membrane and enables mitochondria to uptake calcium. To determine whether MICU1 is affected in vitrified oocytes, we examined MICU1 expression during oocyte vitrification. As shown in Figure 2A, the expression of MICU1 mRNA was unaffected among the three groups. However, Western blot analysis revealed that MICU1 protein levels in the vitrified group were significantly increased compared with that of the control group (*p* < 0.01; Figure 2B,C). These results indicate that MICU1 protein is upregulated in vitrified oocytes.

### 2.3. MICU1 Regulates Mitochondrial Ca^2+^ Uptake

**Figure 3:**
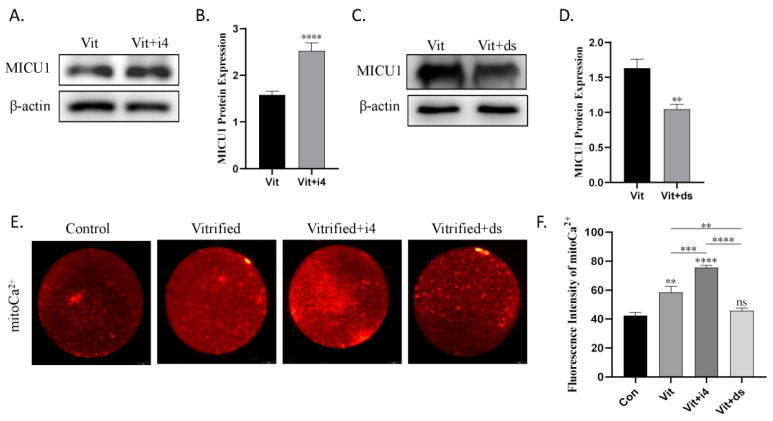
MICU1 stimulates mitochondrial Ca^2+^ uptake in mice vitrified oocytes. (**A**,**B**) Analyses of MICU1 protein expression in vitrified oocytes treated with or without MCU-i4. (**C**,**D**) Analyses of MICU1 protein expression in vitrified oocytes treated with or without DS16570511. (**E**) Fluorescence images showing the mitochondrial Ca^2+^ levels in each group. Scale bar = 20 μm. (**F**) Relative fluorescence intensity for mitochondrial Ca^2+^ in each group. Data in B, D, F are presented as the mean ± SEM. of 3 to 5 independent experiments per group. ns, no significant, ** *p* < 0.01, *** *p* < 0.001, **** *p* < 0.0001. Con, control; Vit, vitrified; Vit+i4, vitrified+MCU-i4; Vit+ds, vitrified+DS16570511.

To determine the role of MICU1 in mitochondrial Ca^2+^ uptake in vitrified oocytes, the MICU1 inhibitor, DS16570511, and MICU1 activator, MCU-i4, were added to the KSOM medium. When vitrified oocytes were supplemented with 2 μM MCU-i4, the level of MICU1 protein was significantly increased (*p* < 0.01; Figure 3A,B). In contrast, the level of MICU1 protein was significantly decreased in the presence of 8 μM DS16570511 (*p* < 0.01; Figure 3C,D). Next, MICU1 inhibition resulted in a decrease in mitochondrial Ca^2+^ uptake (*p* < 0.01; Figure 3E,F), whereas MICU1 activation resulted in an increase in mitochondrial Ca^2+^ uptake (*p* < 0.01; Figure 3E,F). These results illuminate that elevated MICU1 in vitrified oocytes promotes mitochondrial Ca^2+^ uptake.

### 2.4. MICU1 Affects Mitochondrial Membrane Potential and Embryonic Development of Vitrified Oocytes

**Figure 4:**
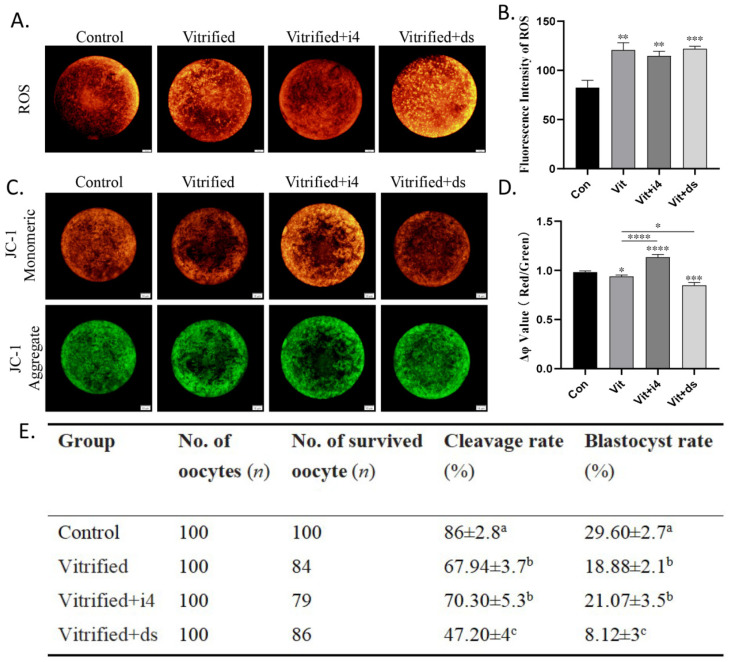
Effect of MCU-i4 or DS16570511 treatment on oocyte quality during oocyte vitrification. (**A**,**B**) Fluorescence images and signal intensity analysis of ROS levels in each group. Scale bar = 10 μm. (**C**,**D**) Fluorescence images and signal intensity analysis of MMP in each grop. Scale bar = 10 μm. (**E**) Survived oocytes: oocytes fron each group that survived with or without differen treatments were used for subsequent parthenogenesis. Cleavage rate: proportion of 2-cell stage embryos from the total number of survived oocytes. Blastocyst rate: proportion of blastocysts from the total number of survived oocytes. Different superscript values mean that the corresponding groups differ significantly from each other (*p* ≤ 0.05). Data in B, D, E are presented as the mean ± SEM. of 3 to 5 independent experiments per group. * *p* < 0.05, ** *p* < 0.01, *** *p* < 0.001,**** *p* < 0.0001. Con, control; Vit, vitrified; Vit+i4, vitrified+MCU-i4; Vit+ds, vitrified+DS16570511.

Next, we determined the effect of MICU1-mediated mitochondrial Ca^2+^ uptake on oocyte quality. ROS levels were unaffected when vitrified oocytes were cultured in the presence of either the MICU1 inhibitor or activator (*p* > 0.05; Figure 4A,B). However, the mitochondrial membrane potential (MMP) of the vitrified oocytes treated with these agents was significantly increased and decreased (*p* < 0.01; Figure 4C,D), respectively. This indicates that MICU1 only positively regulates MMP, but not ROS production. In addition, the development rate of the control group was significantly higher compared with the vitrified group following parthenogenetic activation (*p* < 0.01; Figure 4E), and MICU1 inhibitor treatment significantly reduced the 2-cell and blastocyst rate of the oocytes (*p* < 0.01; Figure 4E). These results indicate that increased MICU1 maintains the developmental potential of oocytes by preserving the MMP after vitrification.

### 2.5. MICU1 Regulates Pyruvate Metabolism in Vitrified Oocytes

**Figure 5:**
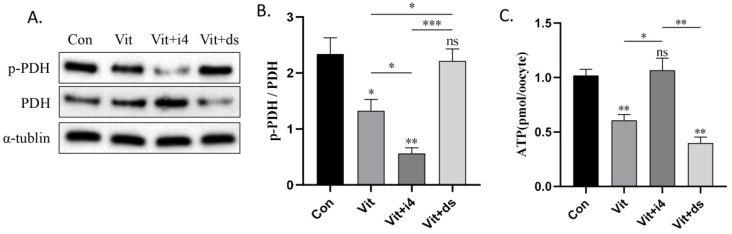
Effect of MCU-i4 or DS16570511 treatment on pyruvate oxidation and energy metabolism in vitrified oocytes. (**A**,**B**) Western blot of p-PDH, PDH and their expression ratio in each group oocytes. (**C**) Average amout of ATP production per oocyte per group. Data in (**B**,**C**) are presented as the mean ± SEM. of 3 to 5 independent samples per group. ns, no significant, * *p* < 0.05, ** *p* < 0.01,*** *p* < 0.001. Con, control; Vit, vitrified; Vit+i4, vitrified+MCU-i4; Vit+ds, vitrified+DS16570511.

In mitochondria, PDH is regulated by calcium ions and activated by dephosphorylation. To determine the effect of MICU1 on pyruvate oxidation in vitrified oocytes, we measured the levels of PDH. The results indicated that p-PDH/PDH was significantly decreased in vitrified oocytes compared with control oocytes (*p* < 0.05; Figure 5A,B). After adding MCU-i4, p-PDH/PDH was further decreased compared with that of the vitrified group (*p* < 0.05; Figure 5A,B), whereas p-PDH/PDH was significantly increased compared with that of the vitrified group after adding DS16570511 (*p* < 0.05; Figure 5A,B).

Consistently, ATP levels were significantly decreased in the vitrified group and were restored by supplementation with MCU-i4 (*p* < 0.01; Figure 5C). Moreover, after adding DS16570511, ATP levels were significantly decreased compared with that of vitrified oocytes. These results suggest that MICU1-mediated mitochondrial Ca^2+^ uptake compensates for energy expenditure during vitrification by activating PDH to enhance pyruvate oxidation.

## 3. Discussion

Our results suggest that MICU1 protein expression was significantly increased in MII oocytes after vitrification. The treatment of vitrified-thawed MII oocytes with MICU1 inhibitor DS16570511 significantly reduced the development rate after parogenetic activation, whereas treatment with MICU1 activator MCU-i4 did not change the development rate after parogenetic activation. DS16570511 decreased the intracellular mitochondrial membrane potential, P-PDH and ATP levels, whereas MCU-i4 increased the intracellular mitochondrial membrane potential, P-PDH, and ATP levels. These differences suggest that MICU1 protein overexpression plays an important role in the maintenance of energy metabolism as well as the developmental competence of vitrified oocytes.

Previous studies proved that mitochondrial Ca^2+^ uptake is mainly accomplished by MCU on mitochondrial intima that shows high selectivity and low affinity for Ca^2+^ [13,14]. MICU1 regulates the rate of mitochondrial Ca^2+^ transport by interacting with MCU [9]. However, this regulation is bidirectional. MICU1 regulates mitochondrial Ca^2+^ uptake by setting a threshold in which Ca^2+^ uptake is blocked when intracytoplasmic calcium levels are low [10]. When intracytoplasmic Ca^2+^ levels increase to the threshold level, Ca^2+^ uptake is increased in coordination with MCU [11]. As a result, the role of MICU1 mainly depends on the mitochondrial Ca^2+^ level it affects: appropriately increased Ca^2+^ concentration in mitochondria can stimulate energy generation and thus have a positive effect on oxidative metabolism [15,16]. Sustained and excessive Ca^2+^ elevation can lead to mitochondrial calcium overload, reactive oxygen species (ROS) increase, and Ca^2+^ homeostasis disorders, leading to cell damage and oxidative stress responses [17,18].

It is known that the increased calcium concentration of oocytes [19,20] and disturbance of calcium oscillation pattern [21] caused by vitrification may be related to zona pellucida hardening, leading to abnormal developmental potential. Currently, vitrification is considered to increase Ca^2+^ concentration in oocytes through two major pathways: Ca^2+^ influx into the extracellular media [22,23] and Ca^2+^ release from the endoplasmic reticulum [24,25]. This will directly increase the concentration of mitochondrial calcium ion [25]. If mitochondrial calcium overload occurs, it will cause mitochondrial oxidative stress and dysfunction, which is not conducive to the subsequent development of oocytes [26].

Accordingly, it is important to identify abnormally expressed MICU1 in oocytes for the study of cryo-damage. Our results showed that the mitochondrial Ca^2+^ level increased significantly and the reactive oxygen species (ROS) level remained unchanged after the vitrified-thawed MII oocytes were treated with 2 μM MICU1 activator MCU-i4. The mitochondrial Ca^2+^ level decreased significantly and reactive oxygen species (ROS) level remained unchanged after the vitrified-thawed MII oocytes were treated with 8 μM MICU1 inhibitor DS16570511. This suggests that MICU1 promotes mitochondrial calcium uptake but does not cause mitochondrial calcium overload after warming.

The energy supply of mammalian MII oocytes is mainly from pyruvate oxidation [27,28]. PDH in mitochondrial intima acts as a bridge between pyruvate aerobic oxidation and the tricarboxylic acid cycle. Its activity is regulated by mitochondrial Ca^2+^ and activated by dephosphorylation [29]. Consistent with previous experimental results, the intracellular ATP levels after warming were significantly reduced by the vitrification of MII oocytes [30,31]. In this study, the phosphorylation level of PDH in mouse oocytes was significantly reduced after vitrification, suggesting that the activity of pyruvate dehydrogenase (PDH) was upregulated. Moreover, treatment with MICU1 activator MCU-i4 significantly increased ATP levels of vitrified oocytes, whereas treatment with MICU1 inhibitor DS16570511 significantly decreased ATP levels of vitrified oocytes. These results further improve the previous view that ATP generation can be realized by mitochondrial Ca^2+^ transport to provide energy for cells [32] and MCU, as the core channel of MCU complex, can let mitochondrial transport and participate in regulating ATP generation [33]. When MICU1, as an important regulatory subunit in MCU complex, plays a synergistic role, the activation of MICU1 is beneficial to ATP production to a certain extent, whereas the inhibition of MICU1 is unfavorable to ATP production. Notably, previous studies have shown that PDH gene knockout oocytes also showed a severely impairment in their energy metabolism and developmental competence [34], just as the MICU1 inhibitor DS16570511 treatment group in this study, suggesting that PDH in mitochondria may be the key target of MICU1’s effect on the energy metabolism of MII oocytes after warming.

In conclusion, the intracellular mitochondrial calcium level of mouse MII oocytes increased after vitrification, which stimulated pyruvate oxidation by decreasing the phosphorylation level of PDH. This process may maintain subsequent oocyte development by compensating for the ATP loss caused by vitrification. The role of MICU1 appears to be crucial to this mechanism. Other roles of MICU1 need to be further studied.

## 4. Materials and Methods

We used two types of MICU1-modulating agents: MCU-i4 (HY-138620, MCE, Birmingham, New Jersey, USA) and DS16570511 (HY-115595, MCE, Birmingham, NJ, USA). In this study, the MICU1 protein level in vitrified oocytes was increased by MCU-i4 and decreased by DS16570511.

### 4.1. Experimental Design and Groups

**Figure 6:**
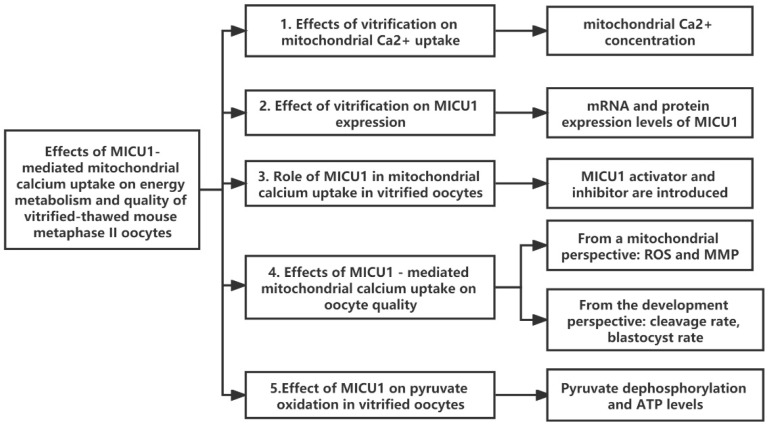
A brief overview of the experiments conducted in this study.

Figure 6 shows a brief overview of the experiments conducted in this study. The experimental groups were as follows. Control: fresh oocytes. Toxicity: oocytes were exposed to the cryopreservation solutions but were not placed in liquid nitrogen. Vitrified: oocytes were exposed to the cryopreservation solutions followed by vitrification in liquid nitrogen. Vitrfied+i4: vitrified-thawed oocytes were exposed to the KSOM medium containing 2 μM MCU-i4 for 2 h at 37 °C. Vitrified+ds: vitrified-thawed oocytes were exposed to the KSOM medium containing 8 μM DS16570511 for 2 h at 37 °C. β-tubulin of vitrified mouse MII oocytes could reassemble at 37 °C for 2 h to form normal metaphase spindle with normal chromatin arrangement, ensuring normal meiosis [35]. So, we chose a two-hour resuscitation or treatment.

### 4.2. Mature Oocytes Collection

Mature oocytes were obtained from 6-week-old female ICR strain mice (Experimental Animal Center of Northwest A&F University, Xi’an, China). Oocyte collection was performed with a few modifications, as described previously [36]. Briefly, the intraperitoneal injection of 10 IU pregnant mare serum gonadotropin (PMSG; Ningbo Hormone, Ningbo, China) was performed for superovulation, and 10 IU human chorionic gonadotropin (hCG; Ningbo Hormone, Ningbo, China) was injected 48 h later. Cumulus-oocyte complexes (COCs) were collected from the tubal enlargement of mice 15 h after hCG injection. The COCs were denuded with 0.2% hyaluronidase (H4272, Sigma, St. Louis, MO, USA) into exposed oocytes at 37 °C. Subsequently, mature (MII) oocytes with a first polar body exhibiting normal morphology were used. Around 30–50 oocytes were obtained per mouse.

### 4.3. Oocyte Vitrification and Thawing

Oocyte vitrification and thawing were performed as described previously [37]. Briefly, modified Dulbecco’s PBS (DPBS) and 3 mg/mL BSA (A1933, Sigma, St. Louis, MO, USA) were used to prepare a mother solution. The vitrification process comprised two basic steps: equilibrium and vitrification. The equilibrium solution comprised 7.5% (*v*/*v*) ethylene glycol (V900208, Sigma, St. Louis, MO, USA) and 7.5% (*v*/*v*) DMSO (D8418, Sigma, St. Louis, MO, USA). The vitrification solution was prepared with 15% (*v*/*v*) ethylene glycol, 171.2 g/L sucrose (S9378, Sigma, St. Louis, MO, USA), 30 g/L Ficoll (F2878, Louis, MO, Sigma, USA), and 15% (*v*/*v*) DMSO. These solutions were used for the equilibrium and vitrification of oocytes. The oocytes were stored in liquid nitrogen for at least 4 weeks. The thawing solution was prepared by dissolving 30 g/L Ficoll and 342.3 g/L sucrose in the mother solution. The diluting solution was prepared by dissolving 171.2 g/L sucrose in the mother solution. The washing solution comprised DPBS.

This study used the Cryotop method. First, the oocytes were placed in equilibrium solution for 5 min, transferred into vitrification solution, and completely rinsed for 1 min. In total, 2–3 oocytes were loaded into each Cryotop straw and immersed into liquid nitrogen. The thawing process involved removing the vitrified oocytes from the liquid nitrogen and immersing them in thawing solution for 1 min at 37 °C. Next, the oocytes were transferred into diluting solution for 3 min and washed twice in washing solution for 5 min. Oocytes were incubated in the KSOM (IVL04, Caisson, Smithfield, UT, USA) medium and resuscitated at 37 °C for 2 h.

### 4.4. Quantitative RT-PCR

Total RNA was isolated from 60 pooled oocytes using Cell-to-Signal^TM^ Lysis Buffer (AM8728, ThermoFisher, Waltham, MA, USA) and then reverse transcribed using the Prime-Script^TM^ RT reagent Kit with gDNA Eraser Kit (RR047A, Takara, Shiga, Japan). gDNA Eraser with strong DNA decomposition activity was used in Kit, and genomic DNA could be removed by 42 °C for 2 min. At the same time, because the reverse transcription reagent contains components that inhibit the activity of DNA decompression enzyme, the samples after the treatment of gDNA Eraser can directly undergo a reverse transcription reaction for 15 min to synthesize cDNA, so the whole process from genomic DNA removal to cDNA synthesis can be quickly completed within 20 min.

Quantitative RT-PCR was performed using the StepOne Plus instrument (Applied Biosystems, Waltham, MA, USA) as reported previously [38]. mRNA levels were quantified by quantitative real-time PCR (qRT-PCR) using TB Green Premix Ex Taq^TM^ II kit (RR820Q, Takara, Shiga, Japan). The TB Green Premix Ex Taq^TM^ II kit supplemented with heat resistant RNaseH, using cDNA as a template for PCR reaction, can well inhibit the damage caused by the residual mRNA in cDNA to the PCR reaction and its buffer has been improved to make the reaction specificity ratio higher. It can inhibit non-specific reactions and can be used for more accurate quantification in a wide range.

**Figure 7:**
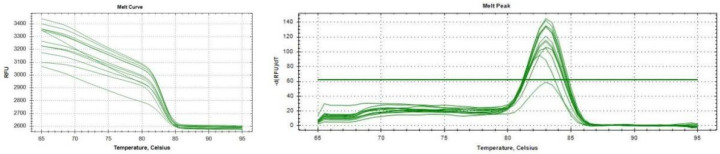
Melt curve and melt peak of flfluorescence quantitative.

β-actin was selected as housekeeping genes through the NCBI software. The relative abundance of MICU1 was expressed as the fold change = 2^−ΔΔCt^. The PCR cycle conditions were as follows: 95 °C for 30 s followed by 40 cycles of 95 °C for 5 s and 60 °C for 30 s. The qRT-PCR data are representative of four independent experiments. Table 1 shows the primer sequences that were designed to span an intron splice site. Figure 7 shows the melt peak and melt curve. It can be seen from the melt peak that the abscissa corresponding to the ordinate peak value is almost the same, indicating that the amplification products of the primers are specific and there is no primer dimer. Considering the difficulty of oocyte collection and the extremely low concentration of cDNA template obtained, we set 40 cycles to improve sensitivity.

### 4.5. Western Blot

Western blot analysis was conducted as described previously [39]. In total 150 control or vitrified oocytes were lysed using the RIPA buffer (P0013B, Beyotime, Shanghai, China) supplemented with a protease inhibitor (P1260, Solarbio, Zhejiang, China) and a protein phosphatase inhibitor. The proteins were separated by 10% SDS-PAGE and then transferred to polyvinylidene fluoride membranes (ISEQ00010, Millipore-sigma, St. Louis, MO, USA). The membranes were blocked with QuickBlock™ Western Blocking Buffer (P0252, Beyotime, Shanghai, China) and incubated with anti-MICU1 mouse monoclonal primary antibody (YM3430, Immunoway, Berkeley, CA, USA), anti-MCU rabbit polyclonal primary antibody (26312-1-AP, Proteintech, Rosemont, Chicago, IL, USA), anti-PDH rabbit monoclonal primary antibody (ab168379, Abcam, Cambridge, UK), anti-PDH (phospho S293) rabbit monoclonal primary antibody (ab177461, Abcam, Cambridge, UK) overnight at 4 °C. After washing, the membranes were incubated with HRP-labeled Goat Anti-Mouse IgG (A0216, Beyotime, Shanghai, China) or HRP-labeled Goat Anti-Rabbit IgG (A0208, Beyotime, Shanghai, China) for 2 h. After washing thrice with TBST for 10 min, the Chemi Doc MP system (Bio-Rad) was used to visualize the immunoreactive proteins. Image J software was used to analyze the gray image level. MICU1 and MCU protein levels were normalized to the housekeeping gene β-actin (66009-1-Ig, Proteintech, Rosemont, Chicago, IL, USA), PDH and p-PDH protein levels were normalized to the housekeeping gene α-tublin (66031-1-Ig; Proteintech, Rosemont, Chicago, IL, USA). Western blotting was repeated five times.

### 4.6. Determination of Mitochondrial Calcium Concentration

To measure mitochondrial calcium concentration, the fluorescent mitochondrial Ca^2+^ probe, Rhod-2 AM (HY-D0989, MCE, Birmingham, NJ, USA), was used. Briefly, around 40 oocytes were incubated with 6 μM Rhod-2 AM in the KSOM medium (14561C, Sigma, St. Louis, MO, USA) for 30 min at 37 °C. After three quick washes with the KSOM medium, the oocytes were imaged using a Nikon fluorescence microscope (Nikon, Tokyo, Japan) at 581 nm. This measurement was conducted four times (n = 20 oocytes for each group).

### 4.7. Determination of Reactive Oxygen Species Levels

The ROS levels in oocytes were determined as described previously [36]. Briefly, around 30 oocytes were transferred into the KSOM medium supplemented with 5% CellROX^®^ Orange reagent (C10443, ThermoFisher, Waltham, MA, USA) for 30 min at 37 °C. Following 3 rapid washes with the KSOM medium, the oocytes were imaged using a Nikon fluorescence microscope at 546 nm. This determination was performed four times (n = 20 oocytes for each group).

### 4.8. Determination of Mitochondrial Membrane Potential

Oocyte mitochondrial membrane potential was detected with the Enhanced Mitochondrial Membrane Potential Assay Kit with JC-1 (C2003S, Beyotime, Shanghai, China). Briefly, about 30 oocytes were transferred into working solution diluted 1:400 with KSOM medium and cultured at 37 °C for 30 min. Following 3 rapid washes with the KSOM medium, the oocytes were imaged using a Nikon BX63 fluorescence microscope. The ratio of red to green fluorescence intensity was calculated to represent mitochondrial membrane potential. The mitochondrial membrane potential was measured four times (n = 20 oocytes for each group).

### 4.9. Determination of ATP Levels

The ATP levels in the oocytes were detected using a commercial ATP assay kit (A095-1-1, Nanjing Jiancheng, Nanjing, China) according to the manufacturer’s instructions. Briefly, 300 μL of hot distilled water containing 60–80 oocytes were transferred into a glass homogenizer and homogenized. The resulting cell suspension was heated in a boiling water bath for 10 min, vortexed, and mixed for 1 min. A colorimetric method was immediately used to determine ATP concentrations using a spectrophotometer (Unico 7200, Franksville, WI, USA). The calculation method of the ATP concentrations in each oocyte (pmol/oocyte) was as described previously [40]. The ATP production was detected three times.

### 4.10. Parthenogenetic Activation and In Vitro Culture of Embryos

Parthenogenesis was performed as described previously [41]. Survival rates were recorded for every 25 mature oocytes treated with or without MICU1 regulator (4 replicates in total). The surviving oocytes were then exposed to calcium-free CZB activation medium containing 10 mM SrCl_2_ and 5 mg/mL Cytochalasin B (4 replicates in total) for 5 h, and then cultured in KSOM at 37 °C in 5% CO_2_ in a chemical atmosphere. Day 0 was the parthenogenetic activation day. Cleavage rate and blastocyst rate were counted on days 1 and 3.5, respectively. Each datum of parthenogenesis was obtained from four experimental repeats.

### 4.11. Statistical Analysis

Statistical analysis was performed using GraphPad Prism 6.0 software. Data are expressed as the mean ± standard deviation of at least three independent experiments. The differences in variables between groups were evaluated by a Student’s t-test (two-tailed). Multiple comparisons between groups were performed using a one-way analysis of variance and a least significant difference test. *P* values < 0.05 were considered statistically significant.

## 5. Conclusions

Vitrified-thawed Mll oocytes promote mitochondrial calcium uptake through the high expression of MICU1 protein. The subsequent elevated mitochondrial calcium concentration stimulates pyruvate oxidation to compensate for ATP loss and maintain developmental competence. 

## Figures and Tables

**Table 1:** Sequence and annealing temperature of flfluorescence quantitative PCR primers used. AT, annealing temperature.

Gene	Sequence (5′–3′)		
	Forward	Reverse	AT (°C)
MICU1	ACCTGGTGAAACCGAAGTGT	TCCAAGGCTGTAGAAGATGC	60
β-actin	TGCTGTCCCTGTATGCCTCT	TTTGATGTCACGCACGATTT	60

## Data Availability

Data is contained within the article.
